# Hernia Cerebri

**Published:** 1844-10-05

**Authors:** 


### HERNIA CEREBRI.

The following causes have been alleged as possibly productive of hernia cerebri:—Firstly, the normal movement of the brain, from the action of the heart and lungs in their unexcited state; secondly, the same movement increased by local congestion, inflammation, and general inflamatory fever; and thirdly, disorganization of the brain, and fluids, the products of inflammation.

